# Neuroprotective effects of saikosaponin-A in ethanol-induced glia-mediated neuroinflammation, oxidative stress via RAGE/TLR4/NFkB signaling

**DOI:** 10.3389/fncel.2025.1625362

**Published:** 2025-08-18

**Authors:** Waqar Ali, Kyonghwan Choe, Min Hwa Kang, Jawad Ali, Hyun Young Park, Abubakar Atiq, Sareer Ahmad, Tae Ju Park, Myeong Ok Kim

**Affiliations:** ^1^Division of Life Science and Applied Life Science (BK21 FOUR), College of Natural Sciences, Gyeongsang National University, Jinju, Republic of Korea; ^2^Department of Psychiatry and Neuropsychology, School for Mental Health and Neuroscience (MHeNs), Maastricht University, Maastricht, Netherlands; ^3^Department of Pediatrics, Maastricht University Medical Center (MUMC+), Maastricht, Netherlands; ^4^Department of Cell Biology, Albert Einstein College of Medicine, Bronx, NY, United States; ^5^Alz-Dementia Korea Co., Jinju, Republic of Korea

**Keywords:** alcohol, neurodegeneration, gliosis, oxidative stress, saikosaponin-A

## Abstract

Chronic use of ethanol leads to psychological and physiological dependence followed by neurodegeneration via glia-mediated neuroinflammation, and oxidative stress. The current study is aimed at the neuroprotective effects of saikosaponin-A against ethanol-induced neurodegeneration. Here, saikosaponin-A 10 mg/kg i.p., for 7 days was used against the ethanol (5 g/kg i.p., for 6 weeks) induced neuroinflammation via RAGE/TLR4 signaling in mouse neurodegenerative model. The immunoblotting and immunofluorescences microscopy results showed that, ethanol activates the glial cells at the level of mice brain. The relative expression of Toll like receptor (TLR4), receptor for advance glycation end product (RAGE), ionized calcium binding adaptor molecules 1 (Iba-1), glial fibrillary acidic protein (GFAP) was upregulated in ethanol-treated mice group. However, expression level of inflammatory biomarkers were downregulated in ethanol + SSA co-treated group. Similarly, our finding revealed that SSA significantly reduced the protein expression level of Phospo c-Jun N-Terminal Kinase (p-JNK), nuclear factor kappa-light-chain-enhancer of activated B cells (NF-kB) and downstream signaling targets in ethanol + SSA co-treated group. SSA also regulates the elevated ethanol-induced oxidative stress via NRF2 and HO-1 proteins. Finally, we analyzed the synaptic and behavioral alteration that was reversed in SSA treated group. Taken together, we concluded that SSA exhibits anti-inflammatory and antioxidant effects against ethanol-induced neurodegeneration.

## 1 Introduction

Alcohol is pharmacologically similar to the hypnotic drugs and can potentially lead to psychological and physiological dependence, which is followed by cognitive and behavioral impairments (Ikram et al., 2019). Among Western, European, and North American societies, the percentage of alcoholics ranges from 2 to 12%. Alcohol abuse may lead to anxiety, depression, and cognitive deficits in experimental rodents (McBride and Li, 1998). Ethanol is one of the alcohols that promote neurological impairment in laboratory experimental rodents. This neurological impairment is associated with neuroinflammation, mitochondrial oxidative stress, and memory impairment (Kamal et al., 2020). Hence, chronic use of alcohol affects different parts of the brain, especially the hippocampal Cornus Ammonis (CA1, CA2, CA3), and Dentate gyrus (DG), as compared to other brain parts of experimental animals (Hedayati-Moghadam et al., 2024). The resident glial cells of central nervous system (CNS) such as microglia and astrocytes can play critical role to protect brain cells from neuroinflammation. Toll-like receptors (TLRs) are a large family and play crucial roles in immunological responses and responses to cellular toxicity (Colonna and Butovsky, 2017). Usually, neuroinflammation is regulated by Toll-like receptor-4 (TLR4), and receptor for advanced glycation end products (RAGE). These receptors are transmembrane receptors bind with the pathogens pattern-associated molecular patterns (PAMPs) as well as to the damage-associated molecular pathogens (DAMPs) of host tissues (Lawrimore, 2018). Previously, it is revealed that TLR4 is a main modulator of ethanol-induced inflammation at the level of CNS. Ethanol-induced neuroinflammation via RAGE and TLR4 leads to synthesis and release of pro-inflammatory molecules, including tumor necrosis factor-alpha (TNF-α), interleukin 1 beta (IL-1β), NF-ĸB, inducible nitric oxide synthase (iNOS), and cyclooxygenase-2 (COX2) in wild-type mice but not TLR4-deficient mice (Pascual et al., 2021). Interestingly, the level of protective interleukine-10 (IL-10) is suppressed by ethanol toxicity, thereby contributing to the promotion of neuroinflammation (Hussain et al., 2017). Elevated interleukins and cytokines cause phosphorylation of NF-kB and JNK proteins leading to mitochondrial oxidative stress which induces synaptic dysfunction (Thakur et al., 2023). Mitochondria are primarily responsible for regulating neurotoxicity exaggerated by alcohol and other related psychological and physiological dependency substances (Pervin and Stephen, 2021). Chronic use of alcohol generates free radicals as well as lipid peroxidation, which ultimately affects the brain and other vital organ systems, especially the liver and brain (Pervin and Stephen, 2021; Albano, 2006). To overcome the consequences associated with the free radicals system, the antioxidant system has a keen interest in neutralizing harmful hallmarks (He et al., 2017). Literature studies revealed that the transcription factor cyclic adenosine monophosphate (cAMP) response element binding protein (CREB) is a target of different signaling pathways and plays a vital role in behavioral processes. Phosphorylation of CREB into p-CREB directly plays a role in synaptic plasticity, as CREB is a transcription factor for the generation of brain-derived neurotrophic factor (BDNF). The CREB, p-CREB/BDNF, is important for neurodevelopment, neurogenesis, and memory impairments (Khakha et al., 2023).

Natural products from different sources, such as plants, animals, and fungi, are the chief entity to prevent and cure different types of diseases (Chopra and Dhingra, 2021). These natural compounds from different sources are drawing interest due to their bioavailability, safety, and efficacy in boosting the body’s natural antioxidant system (Balsano and Alisi, 2009).

Saikosaponin-A (SSA) from *Bupleurum falcatum* L. (Umbelliferae) is a triterpene saponin that has numerous pharmacological properties, including anti-inflammatory, antioxidant, anti-microbial (Sun et al., 2009; Sun et al., 2024), anti-tumor, and potent blocker of cyclooxygenases and lipoxygenases (Zhang et al., 2021). SSA is a compound that inhibits neuroinflammation and oxidation and improves quality of life (Fang et al., 2022; Ali et al., 2019). SSA competitively inhibits inflammatory processing by inhibiting of TLR4, RAGE, nuclear factor-B, Inducible nitric oxide synthase (iNOS), and c-Jun N-terminal kinases (JNK) (Liu et al., 2023; Tan et al., 2025). Based on the therapeutic potential of SSA, we proposed that SSA treatment may inhibit ethanol-induced neurodegeneration and memory impairment in mice via TLR4/RAGE/NF-k-B signaling pathway.

## 2 Materials and methods

### 2.1 Animals

Thirty-two male C57BL/6 N (about 8 weeks old, equally divided into four groups, n = 8 per group) weighing 24-26 g were obtained from Samtako Bio, Osan, South Korea. Female mice fall here in the exclusion criteria because of the estrous cycle, which can alter the normal behavior of mice. The experimental animals were acclimated according to the conditions (temperature, 20 ± 2°C; humidity 40% ± 10%; 12 h light/dark cycle) for 1 week and had free access to food and tap water. The present study was performed by the approved guideline (Approval ID: 125, Code: GNU-200331-M0020) of the Institutional Animal Care and Use Committee (IACUC) of the Division of Applied Life Science, Gyeongsang National University, South Korea.

### 2.2 Chemicals and reagents

Saikosaponin-A (C_42_H_68_O_13_) was obtained from MedChemExpress 9 (Cat. No: HY-N0246) and dissolved in 10% DMSO and 90% normal saline (containing 20% SBE-β-CD). The 2, 7-dichlorodihydrofluorescein diacetate (DCFH-DA) and ethanol, non-denatured, were purchased from Sigma-Aldrich Chemical Co. (St. Louis, MO, United States).

### 2.3 Experimental design

All thirty-two male mice were assigned to the following groups:

- Group 1 (negative control) treated with normal saline i.p.
- Group 2 (positive control) treated with ethanol (5 g/kg i.p., for 6 weeks, daily).
- Group 3 treated concurrently with ethanol (5 g/kg i.p., for 6 weeks, daily) and saikosaponin-A (10 mg/kg i.p. for the last 7 days).
- Group 4 treated with saikosaponin-A (10 mg/kg i.p. for 7 days) only (Ali et al., 2019).

**Figure 1:**
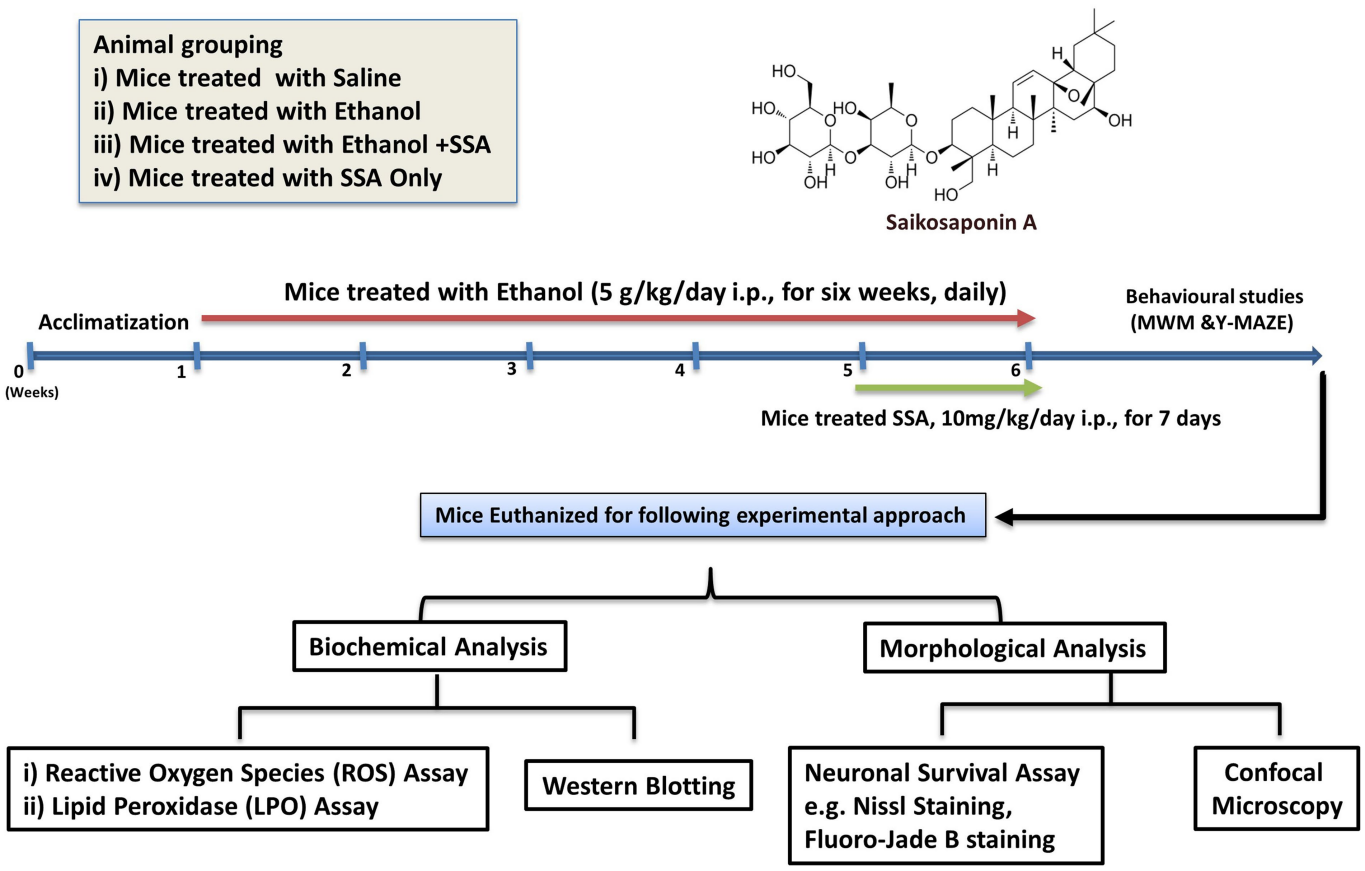
Saikosaponin-A attenuates ethanol-induced neurodegeneration in experimental mice.

After 6 weeks, Y-MAZE (YM) and Morris Water Maze (MWM) behavioral tests were performed to assess spatial memory and learning memory in experimental rodents. The hippocampal tissues were evaluated for oxidation and inflammation. Furthermore, the protein level was assessed by immunoblotting and confocal laser microscopy in the dentate gyrus (DG) and Cornus Ammonis (CA1, CA3). Additionally, cresyl violet and Fluoro-Jade staining were performed in the aforementioned hippocampus areas to study neurodegeneration (Figure 1).

### 2.4 List of antibodies

List of antibodies used for the immunoblotting and immunofluorescences is shown in Table 1.

**Table 1:** List of antibodies used for the immunoblotting and immunofluorescences.

Antibody	Dilution (μl)	Application	Manufacturer	Ref.	Source
TLR4	1:1000	Western Blot/Confocal	Santa Cruz	Sc-293072	Mouse
RAGE	1:1000	Western Blot	Santa Cruz	Sc-80652	Mouse
GFAP	1:1000/1:100	Western Blot/Confocal	Santa Cruz	Sc-33673	Mouse
Iba-1	1:1000	Western Blot	Santa Cruz	Sc-39840	Mouse
p-JNK	1:1000	Western Blot	Santa Cruz	Sc-6254	Mouse
p-NF-KB	1:1000	Western Blot	Santa Cruz	Sc-136548	Mouse
IL-1β	1:1000	Western Blot	Santa Cruz	Sc-12742	Mouse
IL-10	1:1000	Western Blot	Santa Cruz	Sc-8438	Mouse
Nrf2	1:1000/1:100	Western Blot/Confocal	Santa Cruz	12721S	Mouse
HO-1	1:1000/1:100	Western Blot	Santa Cruz	Sc-136961	Mouse
p-Creb	1:1000	Western Blot/Confocal	Santa Cruz	Sc-81486	Mouse
PSD-95	1:1000/1:100	Western Blot/Confocal	Cell Signaling, USA	Sc-71933	Rabbit
SNAP-23	1:1000	Western Blot	Santa Cruz	Sc-374215	Mouse
β-Actin	1:10000	Western Blot	Santa Cruz	Sc-47778	Mouse

All the secondary antibodies were diluted in 1x TBST with 1:10000 μL (v/v).

### 2.5 Behavioral tests

#### 2.5.1 Y-maze

For Y-Maze tests, the entire experimental animal was categorized into four different groups (*n* = 8 per group) and marked properly. The Y-Maze test was primarily used to evaluate spatial working memory, as described in previous studies (Khan et al., 2018). The Y-Maze consists of three equal arms, 50 cm in length, 10 cm in width, and 20 cm in height. All the mice were kept one by one in the middle of the arms to explore the maze with an interval of 8 min and record data with a video tracking system (Panlab HARVARD APPARATUS Smart^®^, Version 3.0.03). The Spontaneous alternation is defined as the consecutive entry of the mice into each three arms in according triplet sets. The alternation behavior (% age) was calculated as successive triplet sets/total number of arm entries –2 × 100 (Ali et al., 2024; Ahmad et al., 2024).

#### 2.5.2 Morris water maze

Another MWM assembly was used to asses learning behavior of experimental rodent as described previously with little modification. The MWM consists of a circular water tank with a height of 40 cm in height and almost 100 cm in diameter. The tank was filled with opaque water (non-toxic white ink was added) up to a depth of 15.5 cm and maintained temperature of 23 ± 1°C. A hidden white platform (20 cm in height, 10 cm in diameter) was placed slightly above the level of filled water in any quadrant. All the experimental mice were trained for 4 days consecutively, each day consisting four numbers of four trials. On day five the probe test was performed, where the mice were allowed to swim freely in the tank for 1 min (the hidden platform was removed). The time spent in the targeted quadrant and non-targeted quadrant and the number of crossing were recorded by a video sensor system attached above (SMART, Panlab Harvard Apparatus, United States) (Khan et al., 2021).

### 2.6 Western blotting

The brain hippocampal tissues (*n* = 4) were homogenized with sufficient amount of PRO-PREPTM extraction solution (iNtRON Biotechnology, Inc., Sungnam, South Korea). After homogenization, the amount of protein was calculated using the Bio-Rad solution (Bio-Rad protein assay kit, Bio-Rad Laboratories, CA, and United States). An equal amount of protein was loaded into 12% gel, and a prestained protein marker (GangNam STAIN™, iNtRON) was used for the identification of the proper molecular weight. The membranes were blocked with 5% (w/v) skim to avoid non-specific binding with primary antibodies for 1 h. Incubate with desired primary antibodies overnight at 4°C. Next, membranes were washed and blocked with secondary antibodies on the source of primary antibodies for 1-2 h. The expression levels were detected by using an ECL reagent with the given protocol (Amersham, Uppsala, Sweden). The results of the bands were quantified via ImageJ software (Ullah et al., 2025).

### 2.7 Immunofluorescence staining

This protocol was carried out as reported previously with minor modifications (Ahmad et al., 2021). The mice’s brains (*n* = 4 per group) were sliced (14-μm) using a CM3050S Cryostat (Leica, Berlin, Germany). The sections were taken on the gelatin-coated slides and stored at freezing temperature. Then, the slides were rinsed with 0.01 M PBS, followed by incubation for 1 h in normal goat serum (2%) and Triton X-100 (0.3%) in PBS. Apply the primary antibodies (diluted in PBS) and incubate overnight at 4°C. Next, the slides were treated with appropriate secondary antibodies (TRITC or FITC-labeled) (Santa Cruz Biotechnology, Dallas Texas United States). To ensure nuclear staining finally, slides were treated with 4′,6-diamidino-2-phenylindole (DAPI). The sample slides were visualized using immunofluorescence microscopy (Fluoview FV 1000 MPE, Olympus, Tokyo, Japan). The obtained valves were evaluated and compared among the experimental groups using ImageJ, and GraphPad Prism 8 (Ullah et al., 2020).

### 2.8 ROS and LPO assays

The reactive oxygen species (ROS) assay was performed with little modification. The main theme of the assay is based on the oxidation of 7-dichlorodihydrofluorescein diacetate (DCFH-DA) to 2, 7-dichlorodihydrofluorescein (DCF). The hippocampal tissue homogenates were diluted in 1 mL of Locke’s buffer (1:20 ratio), 10 mL of DCFH-DA (5 mM), and 0.2 mL of tissue to a final concentration of 5 mg tissue/mL, followed by incubation for 15 min to get a fluorescent DCF. The spectrofluorometric (Promega, Fitchburg, WI, United States) was used to measure the fluorescence of converted DCF an excitation of 484 nm and emission at 530 nm. The blanks were used in parallel to neglect background signals. Lipid peroxidation (LPO) assay is another tool to indicate oxidative stress. The LPO assay kit were purchased from BioVision, San Francesco, CA, United States (Cat#739-100). Here, the free malondialdehyde (MDA) was analyzed in brain hippocampal tissues. The amount of MDA was measured in nmol/mg in each group of hippocampus (Badshah et al., 2019).

### 2.9 Fluoro-Jade B staining

The fluoro-Jade B kit was acquired from Burlington, MA, United States (Cat #AG310, Lot #2159662), and carried as reported with minor modifications. All the slides were immersed in 1% sodium hydroxide and 80% ethanol for 5 to 6 min. Then the slides were treated with 70% ethanol for 2-3 min. Rinsed the slides were distilled water and applied the potassium permanganate solution (0.06%) for 8-10 min, and placed in acetic acid and Fluoro-Jade B solution (0.1 and 0.01% respectively) for 20 min. Finally, the slides were washed with distilled water and apply the coverslips. The slides were visualized using by microscopic technique (FV 1000, Olympus, Tokyo, Japan) and analyzed with ImageJ software (Ahmad et al., 2021).

### 2.10 Cresyl violet staining

Cresyl violet staining was performed to examine morphological study for neuronal survival. The brain-containing slides were washed with filtered PBS (0.01 M) and apply few drops of 0.5% cresyl violet solution for 1 h. Then the slides were washed with distilled water by dipping for 1-2 min. Now dry the slides were different concentrations of ethanol solution (70, 95, and 100%). Furthermore, the slides were dried by applying xylene and covering them with a coverslip using non-florescent mounting media. The effects of Ethanol with or without SSA were visualized under a light microscope and analyzed using ImageJ software, graphically represented via GraphPad Prism 8 software (Ali et al., 2018).

### 2.11 Statistics

The immunoblot and microscopic results were analyzed by using ImageJ software, and normalized by the Shapiro–Wilk normality test. To measure the density in arbitrary units (A. U.) and integrated density to A. U the data were presented as the mean ± SEM for eight mice per group. By using GraphPad Prism 8 software (San Diego, CA, United States), we performed one-way ANOVA with Tukey’s *post hoc* test. The significance is #*p* ≤ 0.05, ##*p* ≤ 0.01, ###*p* ≤ 0.001; *p ≤ 0.05, **p ≤ 0.01, ***p ≤ 0.001.

## 3 Results

### 3.1 Saikosaponin-A inhibits over-activation of glial cells via RAGE/TLR4 receptor

**Figure 2:**
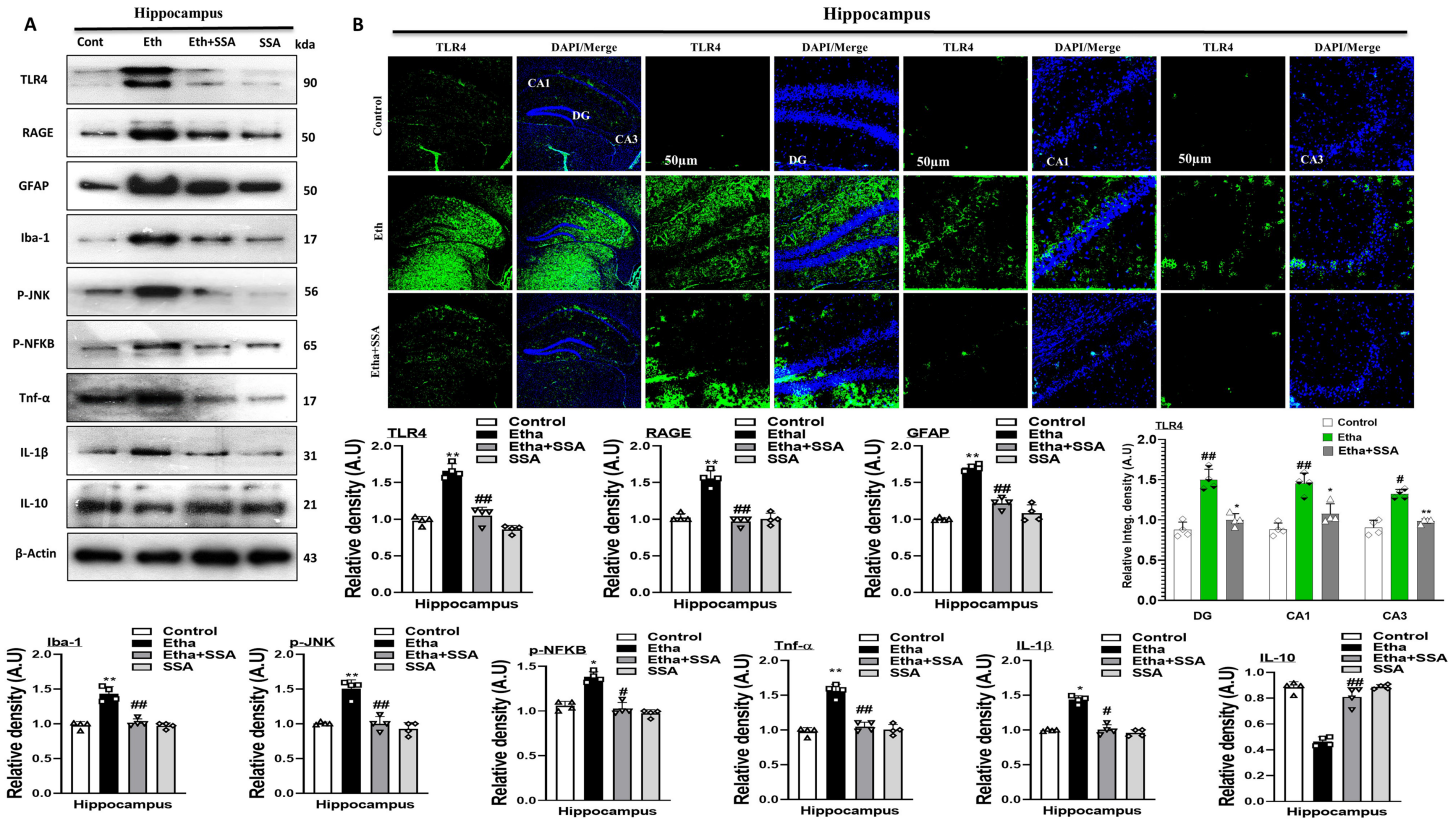
Saikosaponin-A abolishes ethanol-induced glial cell activation and their downstream targets. **(A)** Representative Immunoblotting indicating the protein levels of glial cells biomarkers and level of interleukins and chemokines in the brain (*n* = 4), Image J software was used for quantifying the immunoblots, and graphs were designed using GraphPad Prism. **(B)** Representative Immunofluorescence indicates the fluorescence intensity of mouse hippocampal regions (n = 4), Image J software was used for quantifying the immunofluorescence images and intensity (scale bar 50 μm); graphs were generated using GraphPad Prism. All the data were analyzed in mean ± S. E. M. with respective bar graphs. # Significant difference from the control group, * significant difference from the ethanol-injected mouse group. The significance was: #*p* ≤ 0.05, ##*p* ≤ 0.01, ###*p* ≤ 0.001; *p ≤ 0.05, **p ≤ 0.01, ***p ≤ 0.001.

Mounting literature revealed that glial cell activation is responsible for neuroinflammation and neurological diseases (Yang and Zhou, 2019). Ethanol triggers the glial cells surface receptors such as RAGE/TLR4 and initiates inflammatory processes. Our result showed that ethanol significantly activated gliosis, as evidenced by the remarkably increased expression of RAGE, TLR4, GFAP, and Iba-1 as compared to the control mice. The ethanol + SSA co-treatment group shows remarkable downregulation in the expression levels of above mentioned biomarkers as compared to the expression level in the ethanol-only-treated mice group (Figure 2A).

### 3.2 Saikosaponin-A modulates the expression of p-JNK and its downstream signaling in ethanol-treated mice brain

Studies suggested that Phospho-c-Jun-N-terminal kinase (p-JNK) and nuclear factor kappa-light-chain-enhancer of activated B cells (p-NF-kB) activation is involved in activation and accumulation of different inflammatory mediators at the level of the brain (Kumar et al., 2025). Here, we performed immunoblotting of p-JNK, p-NF-kB, inflammatory mediators (IL-1β), anti-inflammatory mediators (IL-10), and tumor necrosis factor-alpha (TNF-α). Notably, we observe significantly higher protein expression of p-JNK, p-NF-kB, IL-1β, and TNF-α while lower protein expression of IL-10 in the hippocampi of ethanol mice than a control group of mice. Accumulatively, our result proved that SSA remarkably attenuates inflammation and their processing via inhibition of p-JNK, p-NF-kB, and relative inflammatory mediators in different mice groups treated with saline, ethanol and ethanol+ SSA (Figure 2A). In support confocal laser microscopy results also indicated, that ethanol + SSA and SSA only treatment decreases immunoreactivity of glial cells surface receptor TLR4 in the hippocampal areas of mice brain compared to the mice treated with ethanol only (Figure 2B).

### 3.3 Saikosaponin-A regulates ethanol-induced mitochondrial oxidative stress in mice brain

**Figure 3:**
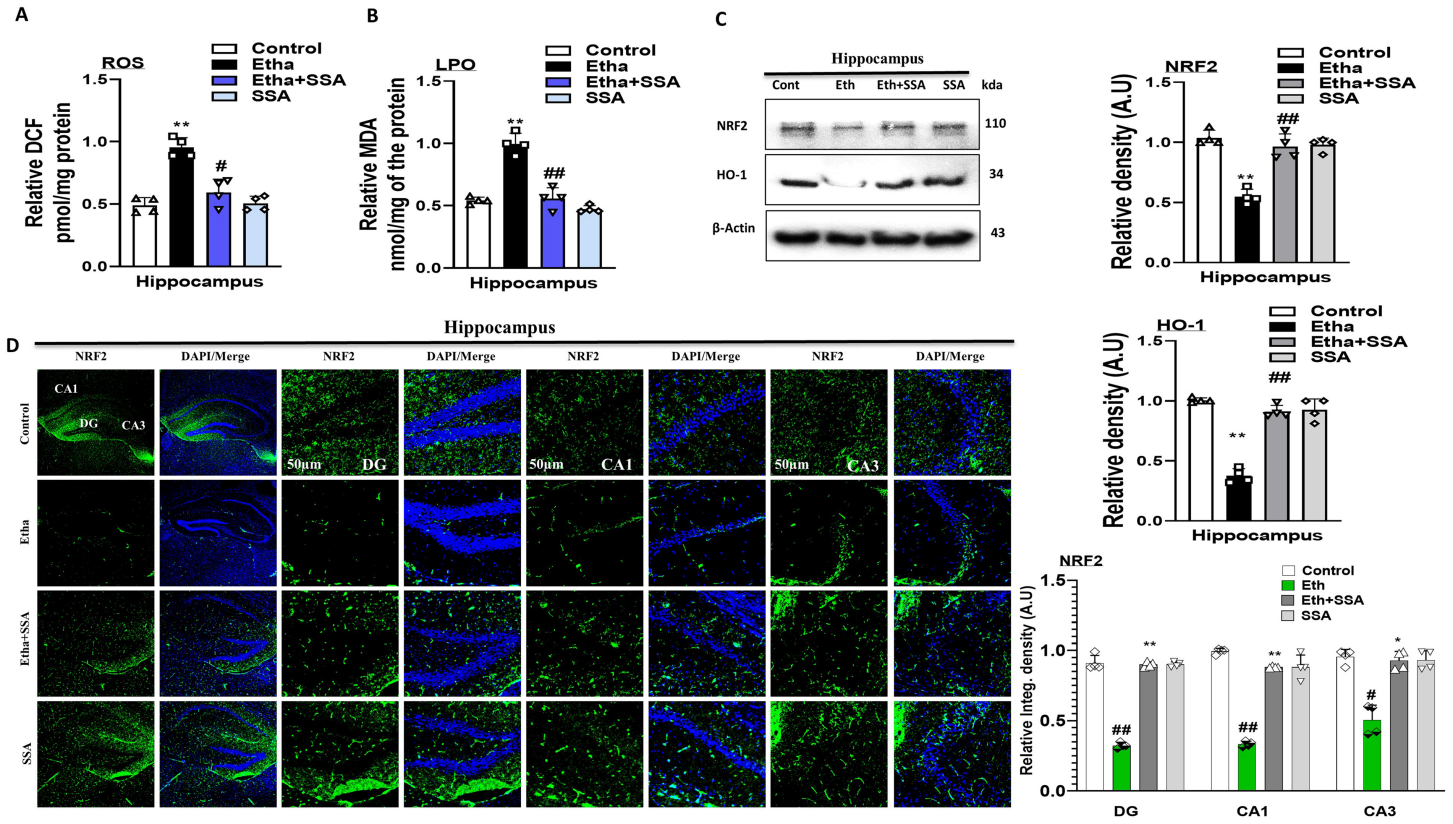
Saikosaponin-A reversed ethanol-induced ROS and LPO levels and downregulation of NRF2 and HO-1 levels in mice brains. **(A,B)** Representative bar graphs of ROS and LPO level in mice brain homogenates (*n* = 4). **(C)** Representative Immunoblots of NRF2 and HO-1 (*n* = 4) antioxidant protein with respective bar graph generated with GraphPad Prism. **(D)** Representing NRF2 Immunofluorescence intensity (scale bar 50 μm) with bar graph (*n* = 4) mice hippocampus. Data were analyzed in mean ± S. E. M. with respective bar graphs. # Significant difference from the control group, * significant difference from the ethanol-injected mouse group. Significance: #*p* ≤ 0.05, ##*p* ≤ 0.01, ###*p* ≤ 0.001; *p ≤ 0.05, **p ≤ 0.01, ***p ≤ 0.001.

Previous studies showed that ethanol is responsible for oxidative stress-mediated neurological diseases such as AD and PD (Sun et al., 2001). To analyze the possible antioxidant effect of SSA against ethanol-induced oxidative stress in mouse brains, the ROS and LPO assays were performed *in vivo*. There is a significant elevation seen in the expression level of both ROS and LPO in ethanol treated group compared to the control group of mice brain hippocampi. Conversely, SSA competitively downregulated the upregulated ROS and LPO expression levels in experimental mice brain (Figures 3A,B).

Similarly, we checked the level of antioxidant protein biomarkers such as NRF2 and HO-1 in mice brains. Western blot findings showed that expression levels of NRF2 and HO-1 were remarkably downregulated in ethanol-treated mice compared to the mice control group. Interestingly, the expression level of the aforementioned biomarkers was upregulated in the ethanol + SSA co-treated group compared to the ethanol-only treated group (Figure 3C). Additionally, we also performed immunofluorescences of NRF2 that strongly support our western blot result. Notably, immunoreactivity of NRF2 was significantly enhanced in hippocampus of ethanol + SSA-treated mice brains compared to the ethanol-treated only group (Figure 3D). Accumulatively, western blot and immunofluorescence analysis showed that SSA is non-toxic to normal mice and non-significant changes were seen between normal saline injected and SSA only treated mice group, and revealed that SSA can reverse ethanol-induced oxidative stress in mouse hippocampus.

### 3.4 Saikosaponin-A treatment improved ethanol-induced synaptic dysfunction

**Figure 4:**
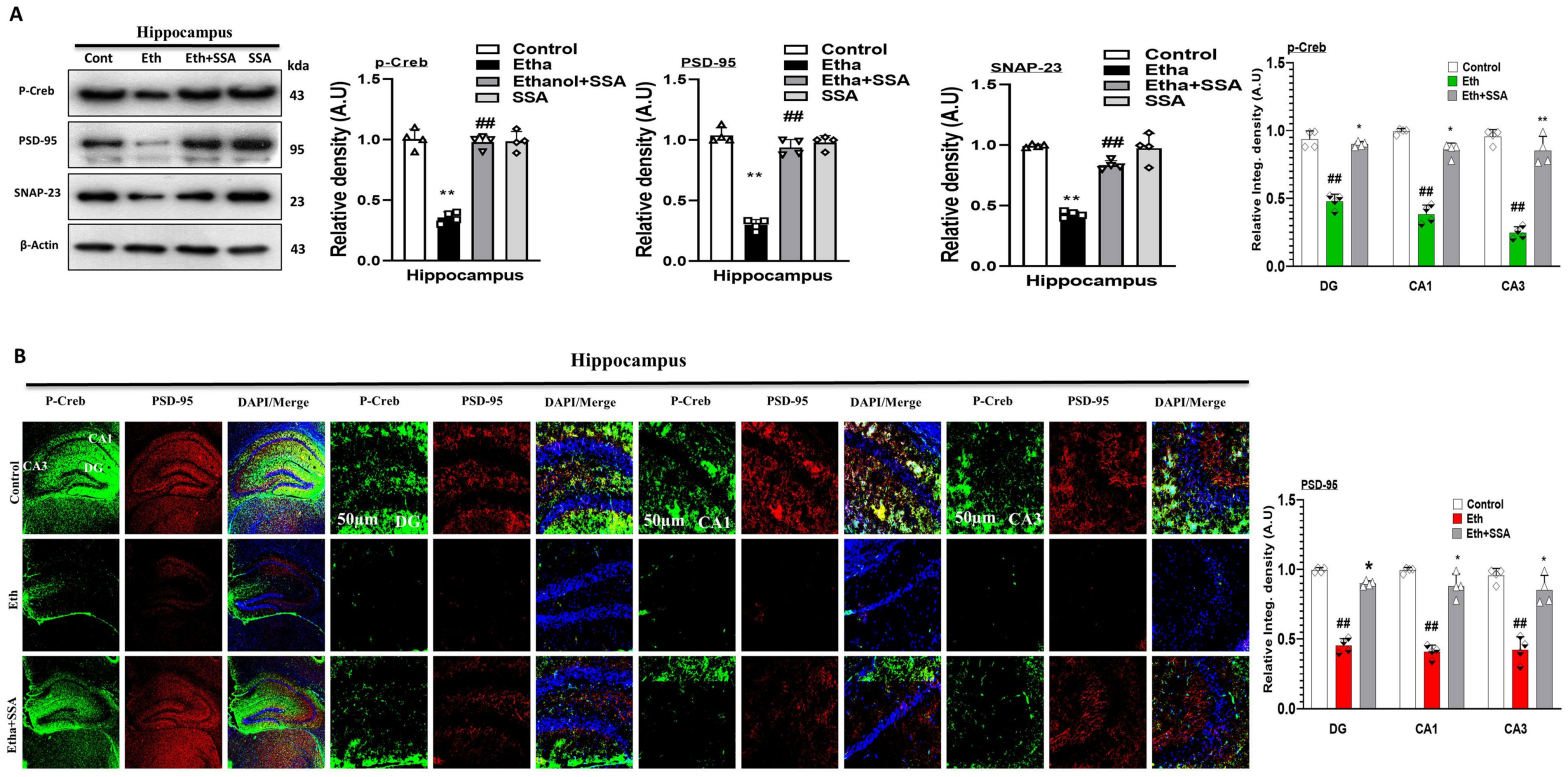
Saikosaponin-A improved synaptic plasticity and reversed cognitive biomarkers. **(A)** Representative western blots p-Creb, PSD-95 and SNAP-23 (*n* = 4). **(B)** Representative co-staining of confocal microscopy results (*n* = 4, scale bar 50 μm) of p-Creb and PSD-95 along with respective bar graphs generated with GraphPad Prism. Data are conveyed as mean ± S. E. M. # Significant difference from the control group, * significant difference from the ethanol-injected mouse group. Significance is #*p* ≤ 0.05, ##*p* ≤ 0.01, ###*p* ≤ 0.001; *p ≤ 0.05, **p ≤ 0.01, ***p ≤ 0.001.

Previously it was reported that there is an elevation in synaptic proteins and genes in ethanol-induced neurodegeneration (Arzua et al., 2024). To analyze the neurotoxic effect of ethanol and the neuroprotective effect of SSA we perform both the immunoblotting and immunofluorescence of mouse brain. Our western blot result remarkably revealed that memory-related protein biomarkers (p-Creb), postsynaptic density protein (PSD-95), and synaptosomal-associated protein 23 (SNAP-23) were downregulated in ethanol only treated group. Conversely, the aforementioned biomarkers were remarkably upregulated in ethanol + SSA co-treated mice hippocampi (Figure 4A). Furthermore, our confocal microscopy result also showed that the immune reactivity of counterstaining of p-Creb and PSD-95 were decreased in the hippocampus of ethanol only treated mice group compared with control mice group. However, in comparison with the ethanol-treated group, the ethanol + SSA co-treated group shows significant increases in the immunofluorescence reactivity of p-Creb and PSD-95 in mice hippocampi (Figure 4B).

### 3.5 Saikosaponin-A enhanced learning, memory, and spontaneous alternation behavior in ethanol-induced memory-impaired mice brain

**Figure 5:**
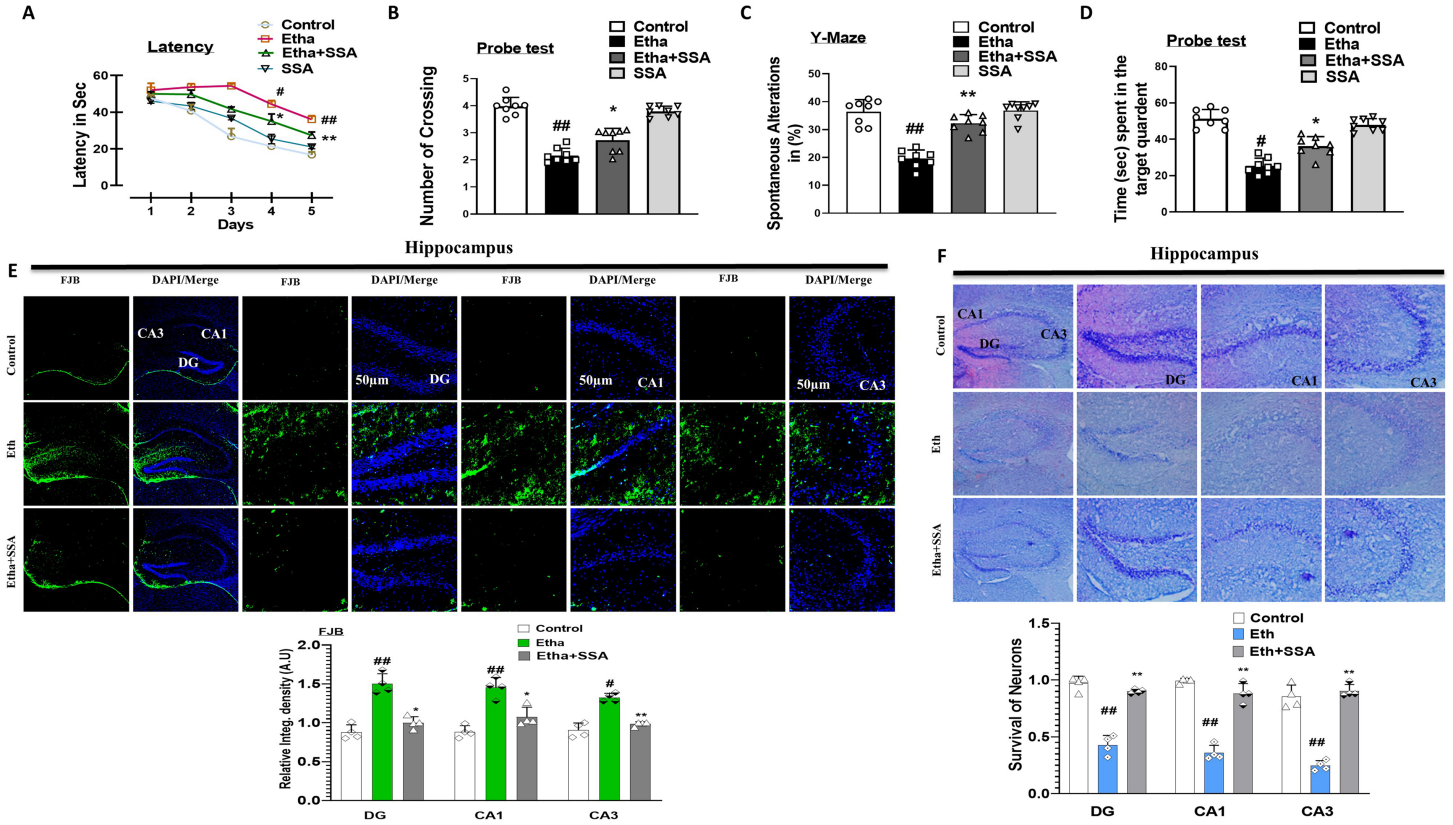
Saikosaponin-A enhanced cognition and retained neuronal survival in mice hippocampal tissues. **(A–D)** Representative bar graphs of behavioral analysis (*n* = 8). **(E)** Representative images of Fluoro-Jade-B staining, and **(F)** representative images of Nissl staining (*N* = 4) with respective bar graphs. Entire data were analyzed in mean ± S. E. M. # Significant difference from the control group, * significant difference from the ethanol-injected mouse group. Significance is #*p* ≤ 0.05, ##*p* ≤ 0.01, ###*p* ≤ 0.001; *p ≤ 0.05, **p ≤ 0.01, ***p ≤ 0.001.

Ethanol affects the behavioral and memory functions in rodents (von der Goltz et al., 2009). To analyze the effect of ethanol and SSA on mice behavior and memory, the Morris water maze (MWM) and Y-maze behavioral tests were performed. In MWM tests, the mean latency to find the hidden platform was decreased in all experimental mice groups except the ethanol-treated group, which exhibited maximum mean latency time as compared to the saline-treated (control) group, showing impairment in spatial learning and memory functions. Similarly, upon completion of all training sessions after 5 days, the hidden platform was removed to perform a probe test. The number of crossing over a platform was increased in the ethanol + SSA treated group as compared to ethanol ethanol-only group. Also, the ethanol + SSA co-treated group mice spent more time in the targeted quadrant than ethanol-only treated mice, which indicated that SSA have ability to reverse ethanol-induced cognitive dysfunction (Figures 5A,B). Next, we performed the Y-maze test to measure spatial working memory function based on spontaneous alteration behavior in percent. An increase in percent (%) spontaneous alteration behavior is a core indicator of improvement in memory functions. We observed that mice treated with ethanol + SSA exhibits high percent (%) spontaneous alteration as compared to the ethanol-only treated group. Finally, our result showed that SSA attenuated short-term memory dysfunction in ethanol-treated mice (Figures 5C,D). Furthermore the Fluoro-Jade B (FJB) and Nissl staining were performed. The FJB positive cells were remarkably decrease with ethanol + SSA co-treated group in comparison of ethanol only group. The Nissl staining notably shows that the level of fragmentation, shrinkage and loss of neuronal cells were seen much greater in the ethanol treated group. These effects were much lower in the ethanol + SSA co-treated group (Figures 5E,F).

## 4 Discussion

The application of natural products from different natural sources such as plants, animals and fungus got keen interest in minimizing the pathology of neurological diseases (Rehman et al., 2019). Saikosaponin-A (SSA) from *Bupleurum falcatum.* L (Umbelliferae) is a triterpene saponin having various pharmacological functions such as anti-inflammatory, antimicrobial, anti-tumor, and antioxidant (Liu et al., 2023). In this study, we investigated the neuroprotective effect of SSA against ethanol-induced neurotoxic effects such as neuroinflammation, oxidative stress-mediated memory impairment, and neurodegeneration in the hippocampi of mouse brain.

Neurodegeneration such as AD and PD is characterized by cognitive impairment and behavioral changes induced by synaptic damage and neuronal damage. As due to the complex pathology and multiple factors, still the exact cause and treatment of AD is unknown and warrant further investigation. Neuroinflammation and mitochondrial oxidative stress are considered major hallmarks that leads to neurodegeneration and cognitive dysfunction (Firdous et al., 2024). Neurological diseases such as AD and PD along with clinical complications affect the life of human beings, reduced courage, and self-independence, and may lead to mortality (Robinson, 2020). Lifestyle modification and proper preventive care for AD and PD are necessary to improve quality of life (Gupta and Gupta, 2020). Different studies showed that various stimuli are there to cause AD and PD like pathologies. Among them, some of the exogenous (ethanol, LPS, and cadmium) (Guo et al., 2024) and endogenous (Aβ-plaque and p-Tau) (Jain and Jain, 2019) stimuli alter cellular homeostatic and metabolic pathways (Althobaiti, 2024). Previous, studies showed that chronic use of ethanol caused gliosis and astrocytosis via RAGE and TLR4 surface receptor-induced pro and inflammatory molecules and different cell types which may also implicated in AD and PD pathogenesis (Pascual et al., 2021). Natural bioactive compounds have a keen interest in controlling and curing of AD and PD-like diseases. SSA is one of the pharmacological bioactive compounds among them used in this study, which attenuates the ethanol-induced neuroinflammation via inhibition of RAGE and TLR4. Similarly, we also investigated the ethanol-induced gliosis (Iba-1 and GFAP respectively) (Saito et al., 2016) which competitively downregulated by SSA treatment in the mice hippocampal tissues. Activation of glial cells leads to phosphorylation of Phospo-c-Jun-N-terminal kinase (p-JNK), nuclear factor kappa-light-chain-enhancer of activated B cells (p-NF-kB), which further responsible for synthesis and release of inflammatory mediators such as tumor necrosis factor (TNF-α) and IL-1β in an ethanol-treated group of mice. These effects were managed remarkably by SSA at the level of hippocampus and prove that SSA exhibits anti-inflammatory properties.

Mitochondrial oxidative stress is another parameter involved in the progression of neurodegeneration and cognitive impairment (Bhat et al., 2015). Progression of oxidative stress in response to ethanol leads to over production of ROS and LPO in mice brains (Hernández et al., 2016). We check the expression level of ROS and LPO in mouse hippocampal homogenates. Notably, our finding suggested that SSA competitively down-regulates the expression level of ROS and LPO and confirms that SSA has an antioxidant effect in mice brains. We also evaluate the antioxidant protein biomarkers, such as NRF2 and HO-1 by western blot and confocal microscopy in the brains of experimental mice. Both techniques showed that SSA has strong antioxidant effects and improves levels of both NRF2 and HO-1 in the ethanol + SSA group in comparison to the ethanol-only treated group.

Structurally neurons consist of synapses and a dendritic spine involved in neuron-to-neuron and neuron-to-effector organ communications. Evidently, cognition and memory impairment are usually co-related with synaptic dysfunction and the number of dendritic spines with active neuronal health (Tzioras et al., 2023). Various pharmacological substances were used to analyze their effects on learning and memory function in different etiological conditions. Here the MWM and YM tests were performed that may SSA improved the cognitive deficits in ethanol treated mice group. Both the learning behavior and spontaneous alteration were enhanced in the ethanol + SSA mice compared to the ethanol-only group and indicated that SSA healed the short-term memory dysfunction in ethanol-injected mice brain. Several studies suggested that ethanol-induced neuroinflammation and oxidative stress accumulatively affect cognitive and motor functions (Baradaran et al., 2021). Our results revealed that SSA enhanced synaptic integrity in the hippocampus of an ethanol-injected AD-like mouse model.

In addition, the FJB and Nissl staining findings strongly support our study. The numbers of FJB-positive cells were significantly downregulated in ethanol + SSA mice compared to the ethanol-injected group. The number of cell shrinkages, damages, and Nissl bodies were much increased in an ethanol-treated group. Conversely, these effects were observed much lower in ethanol + SSA co-treated mouse hippocampal tissues. The use of SSA provides an important insight in ethanol induced neuroinflammation and oxidative stress neurodegeneration, but several limitation are there. The use of animal model does not replicate the human physiological and pathological function such as immunological responses, pharmacokinetics and metabolism. Apart this SSA results provide a strong rationale to check its effects in clinical case study.

## 5 Conclusion

**Figure 6:**
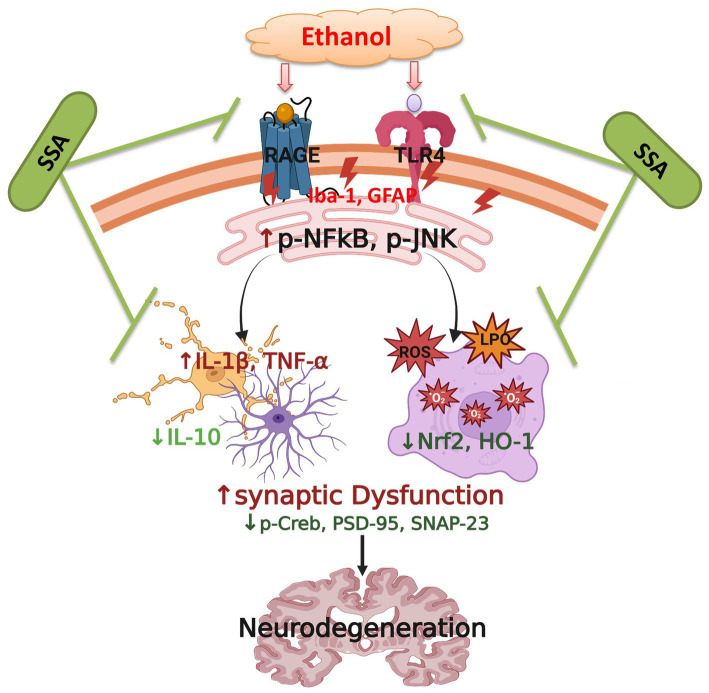
Proposed Mechanism of action of saikosaponine-A abolishing ethanol-induced neurodegeneration in mice brains.

Saikosaponin-A inhibits the glial cells-mediated neuroinflammation in the mouse brain. Competitively SSA also inhibits secretion of TNF-α and IL-1β and mimics the IL-10 from macrophages. Finally, it has been concluded that SSA exhibited anti-inflammatory and antioxidant activity and plays a vital role in the curing of inflammatory and oxidative stress mediated neurodegenerative diseases (Figure 6).

## Data Availability

The original contributions presented in the study are included in the article/Supplementary material, further inquiries can be directed to the corresponding authors.
