# Supplementary material for: Neuroprotective effects of saikosaponin-A in ethanol-induced glia-mediated neuroinflammation, oxidative stress via RAGE/TLR4/NFkB signaling
Source: Front Cell Neurosci. 2025 Aug 18;19:1625362. doi: 10.3389/fncel.2025.1625362 (PMC12399547; doi:10.3389/fncel.2025.1625362)
Supplement: Supplementary file 1 [file Presentation_1.PPTX]

## Slide 1
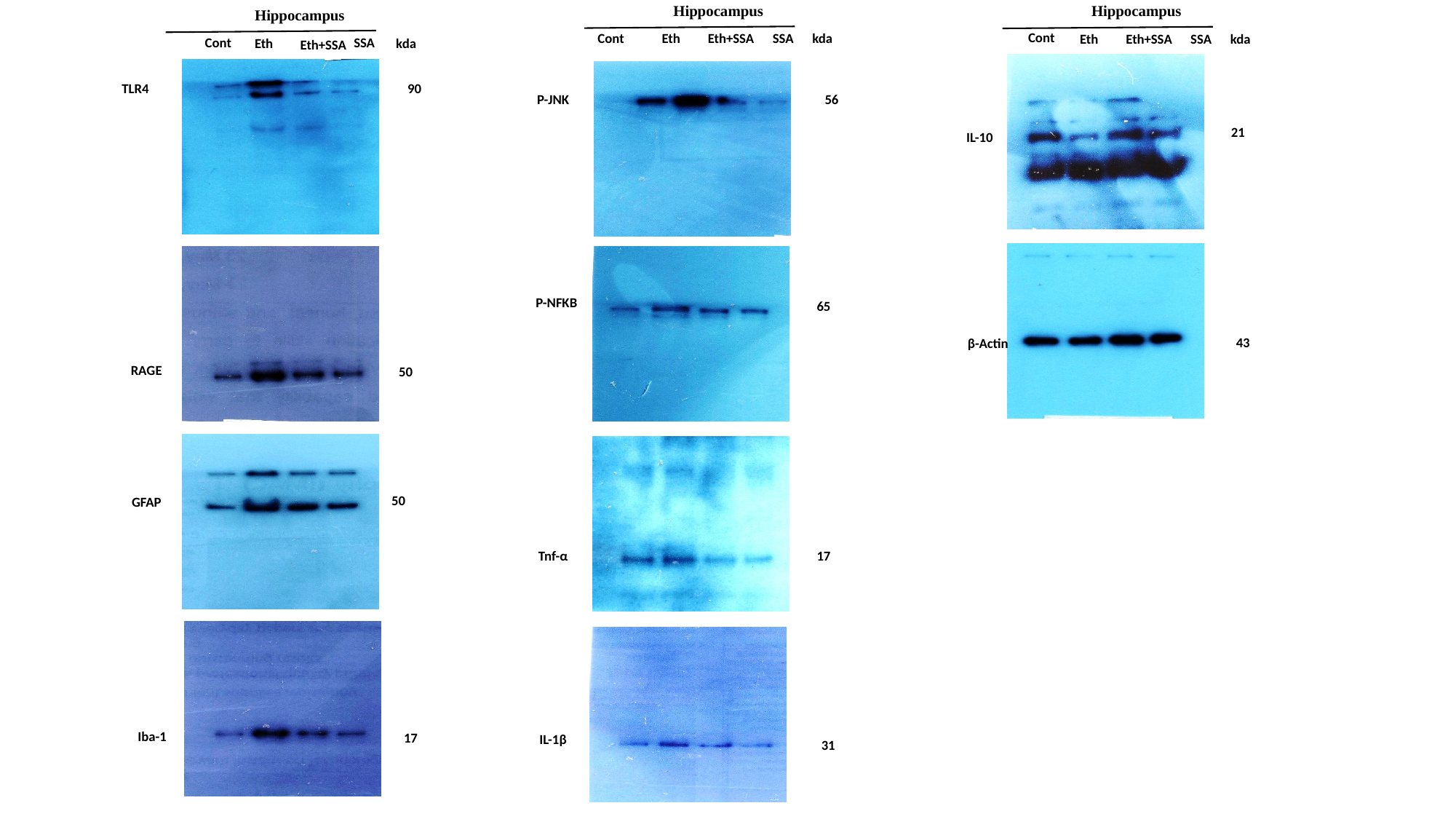

Hippocampus
Hippocampus
Hippocampus
Cont
Cont
Eth
Eth+SSA
SSA
kda
Eth
Eth+SSA
SSA
kda
Cont
SSA
Eth
kda
Eth+SSA
TLR4
90
P-JNK
56
21
IL-10
P-NFKB
65
43
β-Actin
RAGE
50
50
GFAP
Tnf-α
17
Iba-1
17
IL-1β
31

## Slide 2
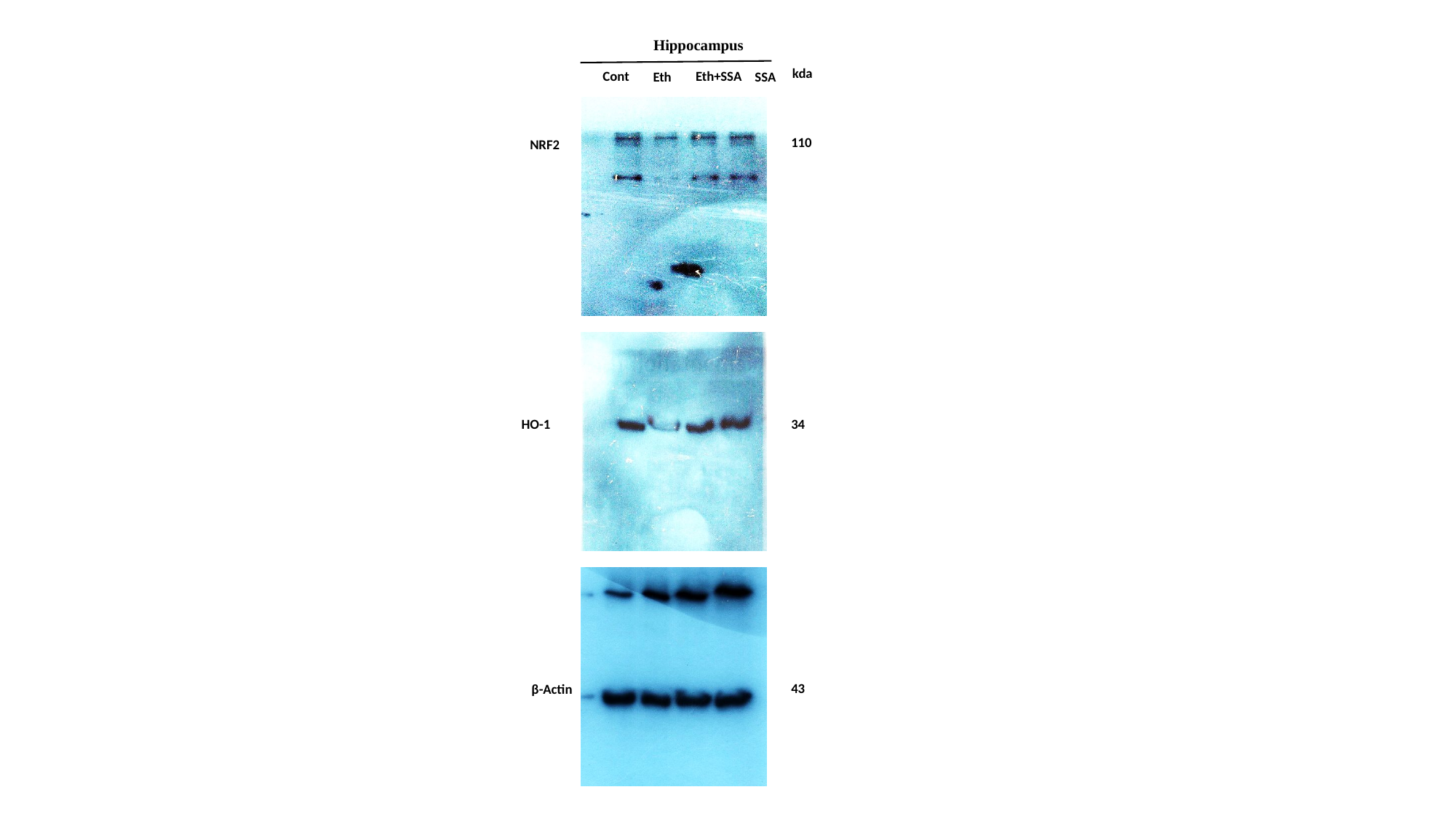

Hippocampus
kda
Cont
Eth+SSA
Eth
SSA
110
NRF2
HO-1
34
43
β-Actin

## Slide 3
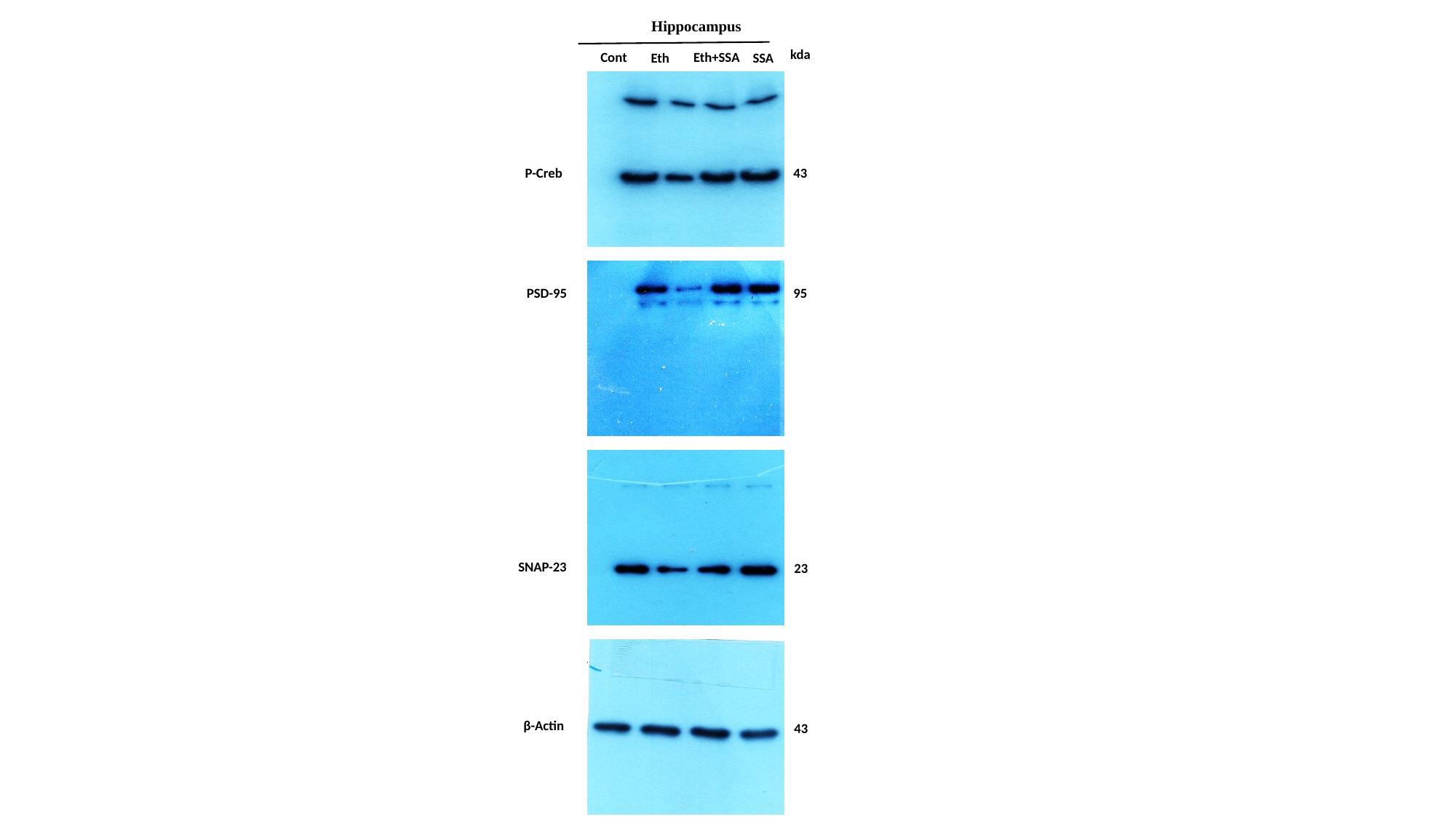

Hippocampus
kda
Cont
Eth+SSA
Eth
SSA
P-Creb
43
PSD-95
95
SNAP-23
23
β-Actin
43
